# Effects of resistance exercise on treatment outcome and laboratory parameters of Takayasu arteritis with magnetic resonance imaging diagnosis: A randomized parallel controlled clinical trial

**DOI:** 10.1002/clc.23439

**Published:** 2020-08-06

**Authors:** Guoce Li, Fenghai Liu, Yan Wang, Meng Zhao, Yancheng Song, Lei Zhang

**Affiliations:** ^1^ Department of MRI Cangzhou Central Hospital Cangzhou China; ^2^ Department of Neurology Cangzhou Central Hospital Cangzhou China

**Keywords:** Birmingham vascular activity score, C‐reactive protein, MRI, resistance exercise, Takayasu arteritis

## Abstract

**Background:**

Elevated tumor necrosis factor‐α (TNF‐α) is correlated with refractory Takayasu arteritis (TA), and resistance exercise have been shown to inhibit TNF‐α.

**Hypothesis:**

We aimed to explore the effect of resistance exercise in the clinical management of TA.

**Methods:**

This clinical trial enrolled a total of 342 acute TA patients, who were subsequently randomized to undergo either resistance exercise or relaxation control twice per week for 12 weeks. The disease activity was defined using the primary outcome of Birmingham Vascular Activity Score (BVAS). Secondary outcomes included levels of plasma TNF‐α and C‐reactive protein (CRP), and the erythrocyte sedimentation rate (ESR).

**Results:**

BVAS scores along with other laboratory parameters obtained from the patients in the resistance exercise group showed a gradual decline throughout the course of the trial. By contrast, outcomes appeared largely unaltered in the relaxation control group patients. Analyses also revealed that plasma TNF‐α displayed strong linear correlations with ESR, BVAS scores, and plasma CRP levels.

**Conclusion:**

Resistance exercise could substantially improve treatment outcomes as well as laboratory parameters in patients with acute TA, probably through decreasing TNF‐α.

## 1 INTRODUCTION

Takayasu arteritis (TA), a chronic granulomatous inflammatory disease, disturbs the aorta and its major branches.^1^, ^2^ TA occurs primarily between the age of 10 to 40, but may also present at an even later age.^3^ TA incidence has increased in Asia, especially among women.^4^ However, conventional therapies cannot effectively attenuate the progressive vascular damages, or establish steady remission of TA.^5^ A long‐term prognosis study revealed that about half of the TA patients, despite intensive medical interventions, still needed percutaneous angioplasty or vascular surgery.^6^ Hence, we are in desperate need for novel therapies that are more effective in improving the treatment outcomes of patients suffering from TA.

Tumor necrosis factor (TNF) is indicated to be an essential regulator in granuloma formation.^7^ Prior investigations have demonstrated, to a certain extent, the clinical efficacy of using TNF inhibitors or antibodies in the treatment against refractory TA.^3^, ^8^, ^9^, ^10^, ^11^, ^12^, ^13^, ^14^ Collectively, these findings suggested that TA treatment with the use of TNF antagonist is promising, although a safer therapeutic agent or approach is needed.

On the other hand, physical exercise and/or training for TA patients is reported to be an effective way of alleviating inflammatory cytokines, such as, TNF,^15^ as well as improving angiogenic functions and physical health.^16^ However, these reports were merely isolated cases, or study of limited number of subjects. Therefore, in order to verify the clinical efficacy of physical exercise on TA symptoms, a large‐scale cohort study is needed to yield statistically reliable results.

Resistance exercise is a type of exercise intervention that requires muscle contraction against external resistance in order to improve the strength and mass of the muscles, as well as the density of the bones.^17^ We conducted this randomized parallel‐controlled clinical trial to determine the efficacy of resistance exercise in treating TA.

## 2 METHODS

### 2.1 Ethics

This trial, conducted from June 2015 to June 2019, used an intend‐to‐treat analysis and complied with the guidelines in the Declaration of Helsinki as well as obtained approval from the Ethics Committee of Cangzhou Central Hospital. All enrolled patients signed informed consents and agreed to our policy regarding data utilization prior to participation.

### 2.2 Patients

This trial enrolled a total of 342 patients with diagnosis of acute TA using the American College of Rheumatology classification criteria^18^: (a) previous magnetic resonance imaging (MRI) results and/or invasive angiography data; (b) C‐reactive protein (CRP) > 5 mg/L; (c) erythrocyte sedimentation rate (ESR) > 30 mm/hours;. All patients were free of tuberculosis as determined by chest roentgenogram. Exclusion criteria were: (d) ongoing systemic infections; (e) breastfeeding or pregnancy; (f) malignancy; (g) chronic or acute liver malfunction; (h) neutropenia with white blood cell density below 4000 cells per mm^3^; (i) thrombocytopenia with platelets less than 120 000 counts per mm^3^; (j) compromised mental or physical abilities; (k) excessive intake of alcohol; (l) record of noncompliance with medical care; (m) with regular physical exercise/training in the previous 6 months. All patients received corticosteroid therapy (Prednisone 1 mg/kg/day and methotrexate 10 mg/week).

### 2.3 Randomization

A total of 294 eligible patients remained and were randomly assigned, with the use of a permuted‐block randomization method stratified according to the basal Birmingham Vascular Activity Scores (BVAS) of these patients,^19^ to either receive relaxation control treatment or resistance exercise training. BVAS score is a clinical index to reflect disease activities, based on signs and symptoms in nine separate organ systems and weighed toward categorical/biochemical and objective evidence of active vasculitis. The BVAS score ranges from 0 to 63, with 0 indicating completely normal.

### 2.4 Intervention

Following the assignment of treatment groups, a machine‐based resistance exercise was conducted twice a week for 12 consecutive weeks guided by experienced therapists. A complete resistance exercise routine took ~1 hour and consisted of eight different progressive machine‐based resistance exercises, namely leg curl, leg extension, leg press, seated row, shoulder external and internal rotation, latissimus pull down, butterfly and butterfly reverse, and shoulder extension and flexion. Each exercise was composed of three sets with 8 to 12 repetitions at a weight of 60% to 80% of one's repetition maximum. If all three sets of an exercise (12 repetitions in total) were completed successfully in three consecutive resistance exercise sessions, the weight would be elevated by at least 5% in the next session. Relaxation control treatment was mainly progressive muscle relaxation without any muscle strengthening or aerobic exercise,^20^ which was also conducted every other week for 12 weeks with each session of ~60 minutes. The resistance exercise as well as the relaxation control intervention was both conducted at the exercise facility inside the hospital.

### 2.5 Anthropometrics

Body weight of patients was measured following an overnight fasting, with no shoes and only light clothing. A digital scale with 0.1 kg accuracy was used for measuring body weight. Body height of patients was determined by a stadiometer with 0.1 cm accuracy. The body mass index (BMI) of each patient was subsequently calculated as: BMI (in kg/m^2^) = body weight/square of height.

### 2.6 Evaluation of outcomes

The disease activity, quantitated using the BVAS,^19^ was defined as the primary outcome. Secondary outcomes included several laboratory parameters, such as, ESR, plasma TNF‐α, and CRP. All results were assessed by physicians unaware of the group assignments. Blood samples were collected from all patients, at the initial enrollment and all the follow‐up visits, to assess the levels of plasma TNF‐α as well as CRP. After collection, blood samples were centrifuged immediately to prevent protein degradation and stored at −80°C within half an hour. TNF‐α and CRP levels in the plasma were determined by Human TNF‐α and CRP enzyme‐linked immunosorbent assay kits (Sigma‐Aldrich) following provided instructions.

### 2.7 Statistical analysis

Data were presented as mean ± SD (SD). The normality of data distribution was determined by the Kolmogorov‐Smirnov goodness‐of‐fit test. To examine normally distributed data, two tailed Student *t* test was employed, whereas for data without normal distribution the Mann‐Whitney test was employed. *P* values less than .05 were regarded as indications of statistical significance. Statistical analyses were conducted using the SPSS software (SPSS Inc.).

## 3 RESULTS

**FIGURE 1:**
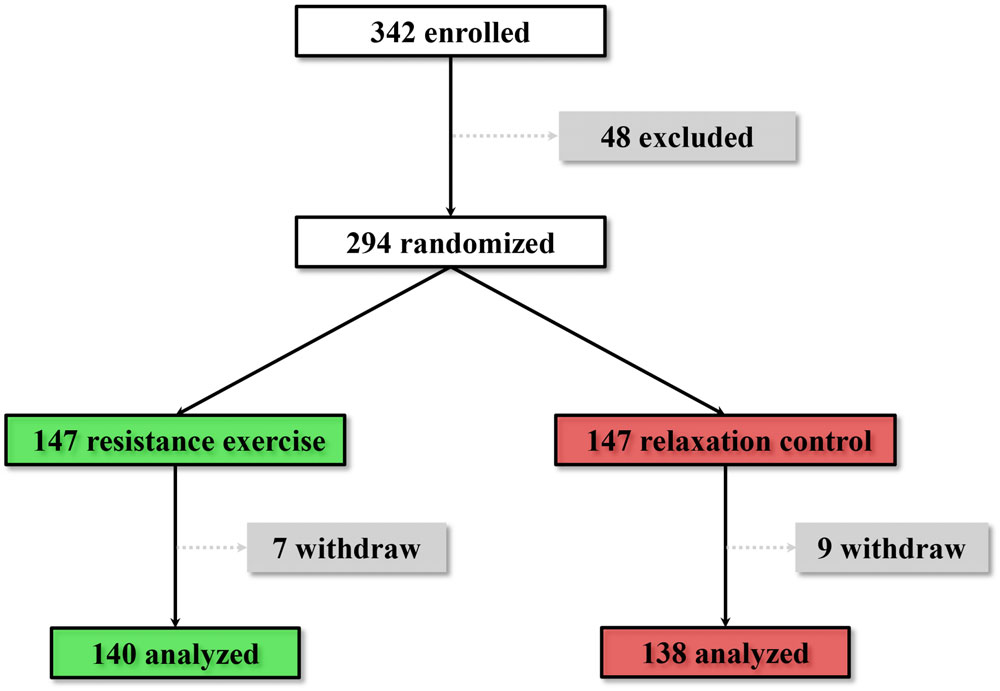
Study design illustration

Design of the current clinical trial was demonstrated as a flow diagram in Figure 1. Among the 342 patients initially enrolled, 48 patients (14.0%) were excluded due to failures to meet the inclusion criteria. The remaining 294 eligible patients participated in this trial, and were evenly and randomly assigned to two treatment groups (n = 147 each group). Following the assignment of groups, 147 patients in the resistance exercise group were instructed under the guidance of experienced therapists to perform a machine‐based resistance exercise twice a week for 12 weeks. The other 147 patients in the relaxation control group performed progressive muscle relaxation without any muscle strengthening or aerobic exercise,^20^ twice a week for 12 consecutive weeks as well.

Throughout the study, 7 (4.8%) patients from the resistance exercise group and 9 (6.1%) patients from the relaxation control group were dropped out from the final analyses because of personal reasons or noncompliance. By the end of the trial, 278 patients, 138 from the relaxation control group and 140 from the resistance exercise group, completed all the follow‐ups as instructed, and their data were presented in the current study.

The characteristics of all patients in both groups at the beginning of the study were compared (Table 1). There was no significant difference regarding the gender, height, age, body weight, BVAS score, TNF‐α, ESR or CRP between patients belonging to the two treatment groups, indicating that the randomization successfully set comparable baselines between the two groups.

**TABLE 1:** Patient characteristics at start of trial

	Resistance exercise (n = 140)	Relaxation control (n = 138)
Male: female	72:68	73:65
Mean age (year, range)	36.6 ± 7.8	37.1 ± 9.3 ^*ns*^
Body weight (kg)	57.4 ± 6.1	56.3 ± 5.7 ^*ns*^
Height (m)	1.72 ± 0.23	1.75 ± 0.26 ^*ns*^
BMI (kg/m^2^)	23.1 ± 2.4	22.9 ± 2.1 ^*ns*^
BVAS score	33 ± 6	32 ± 7 ^*ns*^
TNF‐α (ng/L)	5.98 ± 0.46	6.11 ± 0.31 ^*ns*^
ESR (mm/h)	116.3 ± 8.2	114.8 ± 9.8 ^*ns*^
CRP (mg/L)	40.1 ± 4.2	41.2 ± 3.2 ^*ns*^

*Note*: Data are shown as mean ± SD. *ns* indicates *P* > .05, resistance exercise group vs relaxation control group.

**FIGURE 2:**
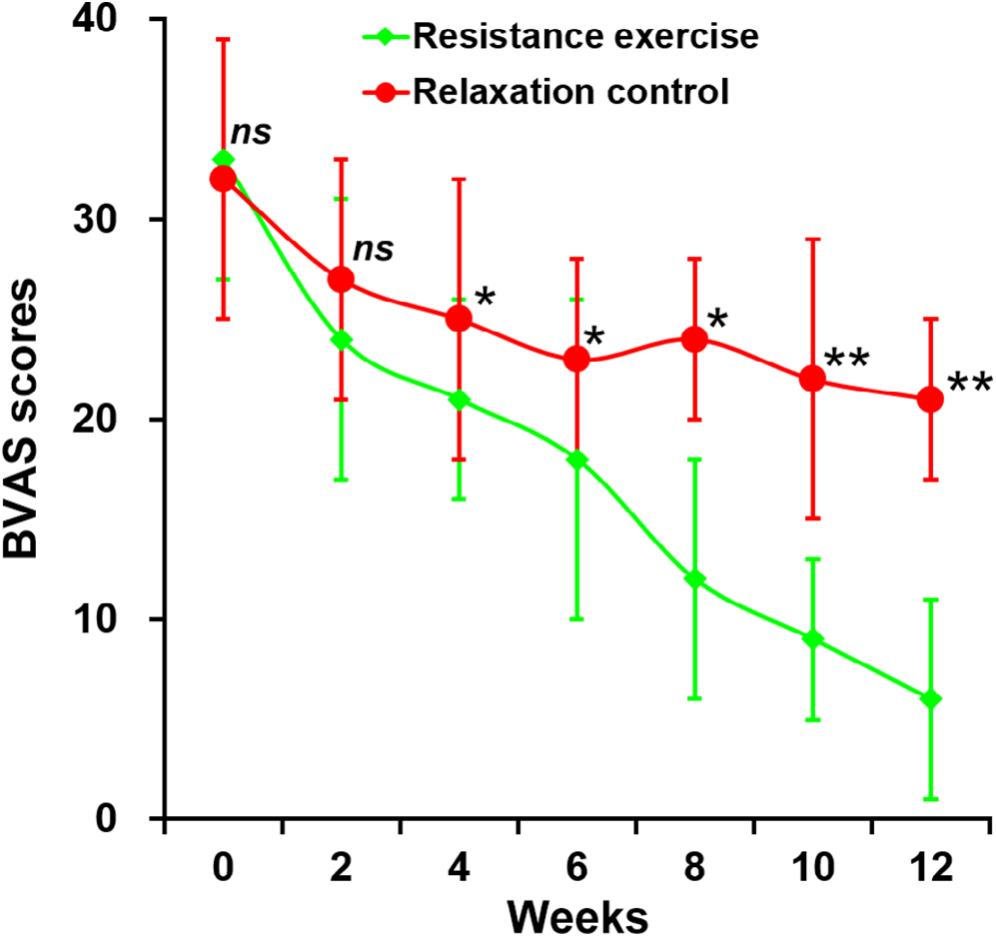
Birmingham Vascular Activity Score scores of eligible patients from the two groups. Data are shown in mean ± SD, **P < .01, *P < .05, *ns* not significant, resistance exercise group vs relaxation control group at indicated follow‐up time points

Patients were revisited every other week for 12 weeks throughout study, and BVAS scores, the primary outcome, from all eligible patients were recorded and analyzed (Figure 2). During the 12 weeks, BVAS scores of patients in the relaxation control group showed a steady albeit slow decline, whereas scores of patients in the resistance exercise group lowered more rapidly. At the start of the study (week 0), and right after the first treatment (week 2), BVAS scores appeared statistically indistinguishable between the two groups (*P* > .05). From week 4 to 8, BVAS score of the resistance exercise group appeared to slightly decline, in comparison with that of the relaxation control group (*P* < .05). From week 10 to the end of study (week 12), BVAS score of patients in the resistance exercise group was found to exhibit a dramatic reduction when compared with that of patients in the relaxation control group (*P* < .01).

**FIGURE 3:**
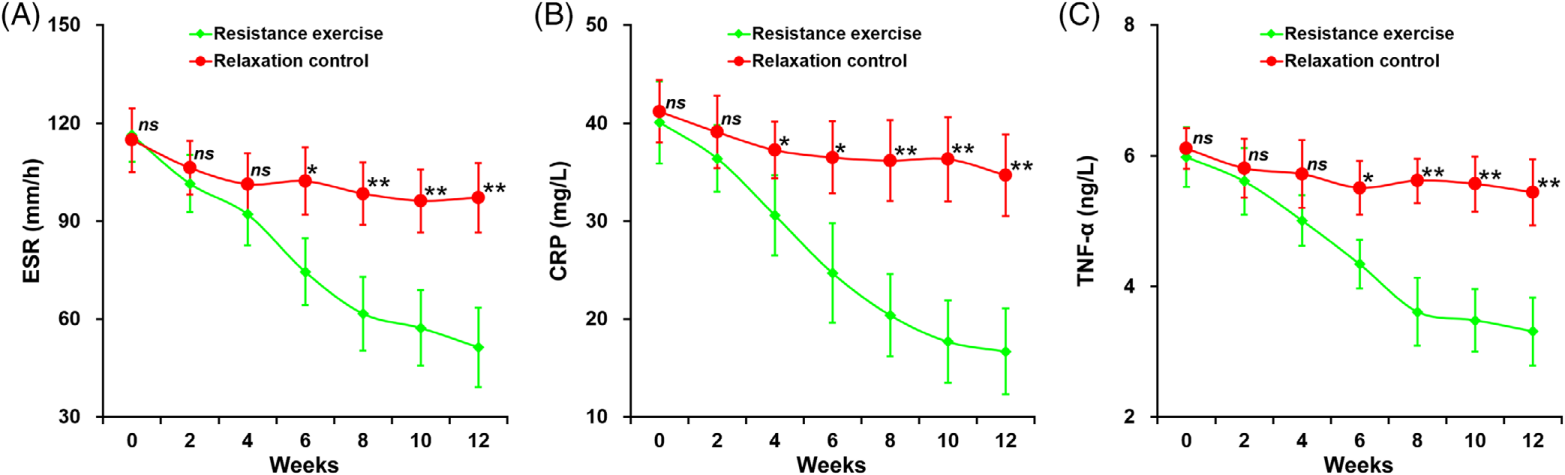
A, Erythrocyte sedimentation rate; B, plasma levels of C‐reactive protein; and C, tumor necrosis factor‐α of eligible patients from the two groups. Data are shown in mean ± SD, **P < .01, *P < .05, *ns* not significant, resistance exercise group vs relaxation control group at indicated follow‐up time points

Laboratory parameters, such as ESR, levels of plasma CRP and TNF‐α, were defined as the secondary outcomes, and all of those parameters displayed a pattern highly consistent to that observed with the BVAS score (Figure 3). All three parameters remained largely unaltered in patients from the relaxation control group during the entire trial, whereas a steady decline of all three was found in patients receiving the resistance exercise. By the end of the study, ESR, plasma TNF‐α, and CRP were all substantially reduced with resistance exercise in comparison to the relaxation control.

Finally, we were curious whether the observed improvement in the primary as well as secondary outcomes of the TA patients was associated with the observed reduction in the plasma TNF‐α level. To this end, we conducted correlation analyses on combined laboratory data from all 278 eligible patients throughout the entire trial (Table 2). We discovered that plasma TNF‐α level indeed had strong correlation with BVAS score (γ = 0.71, *P* = .021), ESR (γ = 0.62, *P* = .037), as well as plasma CRP level (γ = 0.65, *P* = .029). This finding evidently suggested that the decline in BVAS scores as well as laboratory parameters was highly likely associated with the decreased plasma level of TNF‐α.

**TABLE 2:** Linear correlation between TNF‐α and laboratory parameters in all patients

Laboratory parameters	TNF‐α (n = 278)	*P*‐value
BVAS score	γ = 0.71	.021
ESR	γ = 0.62	.037
CRP	γ = 0.65	.029

Abbreviations: BVAS, Birmingham Vascular Activity Score; CRP, C‐reactive protein; ESR, erythrocyte sedimentation rate; TNF‐α, Tumor necrosis factor‐alpha.

## 4 DISCUSSION

The current clinical study of ours is the first to investigate the effect of resistance exercise among a large cohort of TA patients. During the 12 weeks of the entire study, BVAS scores of patients in the resistance exercise group gradually and steadily declined. The reduction in BVAS scores indicated that resistance exercise intervention (60 minutes, twice per week) effectively alleviated TA symptoms. TA can also clinically manifest as inflammation, as indicated by elevation in ESR and plasma levels of CRP.^21^ In the current study, we indeed found reduced ESR and plasma CRP in patients who underwent resistance exercise intervention when compared with those in the relaxation control group. Our findings not only further illustrate the effect of resistance exercise in relieving TA symptoms, but also indicate that the alleviation of TA likely involves the anti‐inflammation process.

TNF‐α, a pro‐inflammation cytokine, is mainly produced by immune cells including natural killer cells, T cells, and macrophages.^22^ TNF‐α participates in a wide range of human diseases including Alzheimer's disease and cancer.^23^, ^24^ In the TA pathogenesis in particular, TNF‐α was shown to exert essential functions in the progression of granulomatous inflammation, a characteristic symptom for this vasculitis.^7^ In patients suffering from active TA, levels of plasma TNF‐α were increased, probably as a result of elevated production of immune cells.^25^, ^26^ Therefore, TNF‐α is a potential therapeutic target for treating TA. Indeed, treatment with TNF antagonists has been reportedly effective in treating many TA patients.^3^, ^11^, ^27^, ^28^ In particular among TA patients resistant to conventional therapies, TNF‐α antagonists, as well as leflunomide and tocilizumab, are considered as new options.^29^ For instance, tocilizumab was reported to be a promising alternative for childhood onset of TA.^30^ In a recent phase 3 trial, tocilizumab exhibited favorable efficacy among patients with refractory TA without any additional safety issue.^31^

Adipose tissue is also involved in TNF‐α production, which can be reflected by increased soluble TNF‐α receptors, IL‐1 or IL‐6 receptor antagonist, and CRP.^32^, ^33^ It is therefore postulated that, during exercise, muscle fibers produce IL‐6 through a TNF‐independent pathway, which then induces other anti‐inflammatory cytokines including IL‐10 and IL‐1ra in the circulation and thereby suppresses the production of the pro‐inflammatory TNF‐α.^34^, ^35^, ^36^ This hypothesis was indeed verified by several studies using both animal and human subjects, in which exercise was shown to inhibit TNF‐α besides other beneficial effects. Exercise decreased plasma levels of TNF through inhibition of TNF production in the spleen of mice.^37^ Resistance exercise was able to decrease oxidative stress and TNF‐α content in the heart of mice with diet‐induced obesity.^38^ In a study involving university students, moderate‐intensity exercise decreased depression and promoted mental health by decreasing TNF‐α.^39^ In a randomized controlled trial among infertile men, exercise intervention attenuated inflammation as evidenced by reduced levels of key pro‐inflammatory cytokines including TNF‐α.^40^ 6 months of resistance exercise could significantly decrease TNF‐α level among elderly subjects.^41^ Intensive exercise stimulation was reported to induce a 6% to 13% decrease of TNF‐α in triathletes.^42^

The potent effect of resistance exercise intervention in reducing TNF‐α observed in our current trial makes it a promising alternative approach in the management of TA. Consistent with the prior exercise intervention studies, we also found a great reduction in the plasma TNF‐α in patients who received 12 weeks of resistance exercise intervention, in comparison to those with relaxation control. Further, we hypothesized that such inhibition on TNF‐α levels by resistance exercise likely contributed to the improved treatment outcomes of TA patients. We indeed discovered that TNF‐α was strongly and positively correlated with BVAS scores, ESR, as well as plasma CRP level, in all eligible patients. This discovery strongly suggests that reduced TNF‐α level could be a contributing factor for the improved therapeutic outcomes in resistance exercise group, and that therapies with anti‐TNF‐α effect could also benefit the outcomes of TA patients.

## 5 CONCLUSION

In conclusion, we hereby provide the first line of statistically significant data supporting the efficacy of resistance exercise, likely attributable to the inhibition of the pro‐inflammatory cytokine TNF‐α, in the clinical management of TA, However, in our current pilot study, the impact of resistance exercise was evaluated over only 12 weeks, a relatively brief period, in only ethnic Chinese patients. The promising results of this trial should give rise to future studies of longer term and with inclusion of patients from more diverse ethnical backgrounds.

## CONFLICT OF INTERESTS

The authors declare no potential conflict of interests.

## ACKNOWLEGMENT

This work was funded by 2017 Cangzhou Science and Technology Research and Development Guidance Plan Project (172302020).
